# Dose-dependent LSD effects on cortical/thalamic and cerebellar activity: brain oxygen level–dependent fMRI study in awake rats

**DOI:** 10.1093/braincomms/fcae194

**Published:** 2024-06-04

**Authors:** Ashley Ghaw, Alisha Chunduri, Arnold Chang, Richard J Ortiz, Milena Kozlowska, Praveen P Kulkarni, Craig F Ferris

**Affiliations:** Center for Translational Neuroimaging, Northeastern University, Boston, MA 02115, USA; Center for Translational Neuroimaging, Northeastern University, Boston, MA 02115, USA; Center for Translational Neuroimaging, Northeastern University, Boston, MA 02115, USA; Department of Chemistry and Biochemistry, New Mexico State University, Las Cruces, NM 88003, USA; Center for Translational Neuroimaging, Northeastern University, Boston, MA 02115, USA; Center for Translational Neuroimaging, Northeastern University, Boston, MA 02115, USA; Department of Psychology and Pharmaceutical Sciences, Northeastern University, Boston, MA 02115, USA

**Keywords:** lysergic acid diethylamide, cerebellar nuclei, BOLD resting-state functional connectivity, 5-HT_2A_ receptor

## Abstract

Lysergic acid diethylamide is a hallucinogen with complex neurobiological and behavioural effects. This is the first study to use MRI to follow functional changes in brain activity in response to different doses of lysergic acid diethylamide in fully awake, drug-naive rats. We hypothesized that lysergic acid diethylamide would show a dose-dependent increase in activity in the prefrontal cortex and thalamus while decreasing hippocampal activity. Female and male rats were given intraperitoneal injections of vehicle or lysergic acid diethylamide in doses of 10 or 100 *µ*g/kg while fully awake during the imaging session. Changes in blood oxygen level–dependent signal were recorded over a 30-min window. Approximately 45-min post-injection data for resting-state functional connectivity were collected. All data were registered to rat 3D MRI atlas with 173 brain regions providing site-specific increases and decreases in global brain activity and changes in functional connectivity. Treatment with lysergic acid diethylamide resulted in a significant dose-dependent increase in negative blood oxygen level–dependent signal. The areas most affected were the primary olfactory system, prefrontal cortex, thalamus and hippocampus. This was observed in both the number of voxels affected in these brains regions and the changes in blood oxygen level–dependent signal over time. However, there was a significant increase in functional connectivity between the thalamus and somatosensory cortex and the cerebellar nuclei and the surrounding brainstem areas. Contrary to our hypothesis, there was an acute dose-dependent increase in negative blood oxygen level–dependent signal that can be interpreted as a decrease in brain activity, a finding that agrees with much of the behavioural data from preclinical studies. The enhanced connectivity between thalamus and sensorimotor cortices is consistent with the human literature looking at lysergic acid diethylamide treatments in healthy human volunteers. The unexpected finding that lysergic acid diethylamide enhances connectivity to the cerebellar nuclei raises an interesting question concerning the role of this brain region in the psychotomimetic effects of hallucinogens.

## Teaser

Ferris *et al*. gave lysergic acid diethylamide to awake rats during MRI scanning. They found a dose-dependent decrease in brain activity followed by an increase in functional connectivity between the thalamus and somatosensory cortices. There was an increase in connectivity to the cerebellar nuclei, previously unreported in clinical studies.

## Graphical Abstract

**Graphical Abstract:**
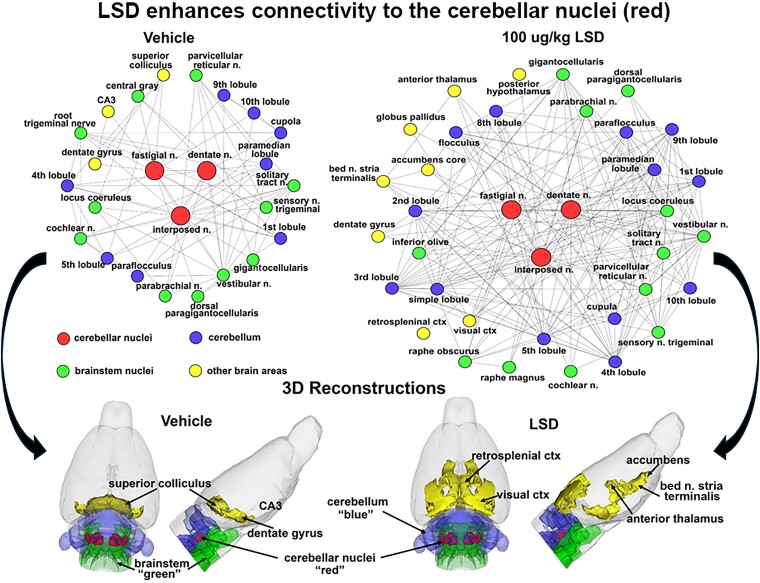

## Introduction

Lysergic acid diethylamide (LSD) was discovered in 1943 and rapidly proliferated into the foremost drug of interest for research in psychiatry.^1^ Research on LSD was halted in the 1970s, but reinitiated in the 1990s, with the first modern clinical trials being published within the last two decades.^2^ Contemporary research continues to focus on the use of hallucinogens for the treatment of psychiatric disorders such as depression.^3^ However, the underlying effects of LSD on the activity of brain neurocircuitry and how these effects influence behaviour are not well understood.

Pharmacological MRI (phMRI) is a non-invasive technique used to analyse alterations in brain function and connectivity in response to CNS therapeutics. Several functional imaging investigations have focused on administering LSD to healthy volunteers in an attempt to map specific pathways associated with the psychedelic experience.^4-6^ These studies share a similar design, involving the administration of a single dose of LSD orally or intravenously outside the scanner. Subjects are then imaged for changes in resting-state functional connectivity (rsFC) compared to data collected after receiving a placebo, typically 60–150 min later. Generally, these studies reveal consistent alterations in brain activity across various major brain regions. Notably, there is an observed increase in thalamo-cortical connectivity, which may explain visual hallucinations, and a reduction in activity in the parahippocampus, potentially contributing to the dysregulation of the resting state and the preservation of the self or consciousness as currently understood.^4,7^ When studying its effects on sociability and affect, further research suggests that LSD operates through a 5-HT_2A_-dependent mechanism.^6^ However, LSD is a promiscuous drug believed to operate through many circuits and signalling pathways and necessitates further investigation to better understand its multifaceted effects.^8-10^

Studying the neurobiological effects of LSD on awake rodents is critical when comparing data between animal models and humans.^11^ Given the sensitivity of the brain to different doses of LSD and the activation of numerous signalling pathways, phMRI offers a method for screening LSD for its global activity and effect on different integrated neural circuits independent of its mechanism of action.^12^ To date, there are no phMRI studies on LSD in awake animals. However, other hallucinogens, e.g. 3,4-methylenedioxymethamphetamine and S-ketamine, have been studied.^13,14^ For example, Kawazoe *et al*.^14^ reported an inverse dose-dependent change in blood oxygen level–dependent (BOLD) signal across the cortex, thalamus and hippocampus in response to S-ketamine given to awake male and female rats during the scanning session.

This study used phMRI in awake male and female rats to evaluate the effect of LSD on brain activity in drug-naive animals. The hypothesized increase in BOLD signal to the thalamus and sensorimotor cortex was not realized with the acute presentation of drug. To the contrary, there was a dose-dependent increase in negative BOLD. Only after 45 min, and then using rsFC, did we see an increase in connectivity between thalamus and cortex. Moreover, there was a significant increase in connectivity to the cerebellar nuclei, a finding that raises question about the role of the cerebellum in the psychotomimetic effects of hallucinogens and the active symptoms of schizophrenia.

## Materials and methods

### Animals

Adult male (*n* = 18) and female (*n* = 18) Sprague–Dawley rats were purchased from Charles River Laboratories (Wilmington, MA, USA). Animals were housed in Plexiglas cages (two per cage) and maintained in ambient temperature (22–24°C). Animals were maintained on a reverse light–dark cycle with lights off at 0900 h and studied during the dark phase when they are normally active. All experiments were conducted between 1000 and 1800 h to avoid the transitions between the light–dark cycles. Food and water were provided *ad libitum*. All animals were acquired and cared for in accordance with the guidelines published in the National Institutes of Health Guide for the Care and Use of Laboratory Animals. All methods and procedures described below were pre-approved by the Northeastern University Institutional Animal Care and Use Committee under protocol number 20-0626R. Northeastern University Animal Care and Use Program and housing facilities are fully accredited by AAALAC International. The protocols used in this study followed the ARRIVE guidelines for reporting *in vivo* experiments in animal research.^15^ Animals were monitored daily over the duration of the study for general health, food and water consumption. A 15% loss in body weight was set as a humane end-point.

### LSD preparation and administration

LSD was acquired through the National Institute on Drug Abuse and distributed by the Research Triangle Institute. On the day of imaging, LSD was prepared in sterile saline (0.9% NaCl), with a final concentration of 100 *µ*g/kg. To deliver drug remotely during the imaging session, a polyethylene tube (PE-20), ∼30 cm in length, was positioned in the peritoneal cavity. The range of doses of LSD were taken from the literature.^9^ Based on body weight, rats were randomly assigned to one of three experimental groups: (i) vehicle (Veh); (ii) 10 *µ*g/kg; and (iii) 100 *µ*g/kg of LSD. Each group consisted of 12 rats divided equally between males and females. Due to motion artefact, five rats were excluded from the study: two male rats and one female rat from the 10 *u*g/kg group and two male rats from the Veh group.

### Acclimation for awake imaging

To mitigate the stress associated with head restraint, rats underwent an acclimation protocol to familiarize them with the restraining system, which consisted of a head holder and body tube. This system was designed to include a cushioned head support, eliminating the need for ear bars and, in turn, reducing discomfort to the animals while minimizing any unintended motion artefacts. These acclimation sessions were conducted daily for five consecutive days. During these sessions, rats were briefly anaesthetized with 1–2% isoflurane for placement into the restraining system. Their forepaws were fastened using surgical tape.

Once fully conscious, the rats were positioned within an opaque black box, essentially a ‘mock scanner’, for 60 min. Inside the mock scanner, a tape recording of the MRI pulse sequence was played to simulate the environment of the magnet bore and the imaging protocol. Under these conditions, there is a significant decrease in respiration, heart rate, motor activity and plasma corticosterone levels when comparing the first and last acclimation sessions, as reported by King *et al*.^16^ This reduction in autonomic and somatic indicators of arousal and stress contributed to the enhancement of signal resolution and image quality.

### Image acquisition

Five to six rats were imaged in a day. Each day had a mix of the different experimental groups known by all the investigators. Rats were scanned at 300 MHz using a quadrature transmit/receive volume coil built into the rat head holder and restraining system for awake animal imaging (Ekam Imaging, Boston, MA, USA). A video of the rat preparation for imaging is available at www.youtube.com/watch?v=JQX1wgOV3K4. The design of the coil provided complete coverage of the brain from olfactory bulbs to brainstem. Radio frequency signals were sent and received with a quadrature volume coil built into the animal restrainer (Ekam Imaging, Boston, MA, USA).^11^ Imaging sessions were conducted using a Bruker BioSpec 7.0 T/20 cm USR horizontal magnet (Bruker, Billerica, MA, USA) and a 2 T/m magnetic field gradient insert (ID = 12 cm) capable of a 120-*μ*s rise time. At the beginning of each imaging session, a high-resolution anatomical dataset was collected using a rapid acquisition with relaxation enhancement (factor 8) pulse sequence [25 slices, 1 mm; field of view, 3.0 cm^2^; data matrix, 256 × 256; repetition time, 3 s; echo time (TE), 12 ms; effective TE, 48 ms; number of excitations, 3; acquisition time, 4.48 min; and in-plane resolution, 117.2 *μ*m^2^]. Functional images were captured using a multi-slice half-Fourier acquisition single-shot turbo spin-echo pulse sequence. This involved collecting 22 slices, each 1.1 mm thick, with the same field of view of 3.0 cm². The data matrix was 96 × 96, with a repetition time of 6 s, TE of 3.75 ms, and an effective TE of 22.5 ms. The acquisition took ∼35 min, with an in-plane resolution of 312.5 *μ*m². It should also be emphasized that high neuroanatomical fidelity and spatial resolution are critical in identifying distributed neural circuits in any animal imaging study. Many brain areas in a segmented rat atlas have in-plane boundaries of less than 400 *μ*m^2^ and may extend for over 1000 *μ*m in the rostral/caudal plane. With the development of a segmented, annotated 3D MRI atlas for rats (Ekam Solutions, Boston, MA, USA), it is now possible to localize functional imaging data to precise 3D ‘volumes of interest’ in clearly delineated brain areas. This spatial resolution was sufficient to identify the bilateral habenula, with approximately four to five voxels on each side, but not to differentiate between the lateral and medial habenula.

### Statistical analysis

The functional MRI data analysis consisted of three main steps: pre-processing, processing and post-processing. All these steps were executed using SPM-12 (available at https://www.fil.ion.ucl.ac.uk/spm/) and in-house MATLAB software. In the pre-processing stage, several operations were performed, including co-registration, motion correction, smoothing and detrending. Co-registration was carried out with specific parameters: quality set at 0.97, smoothing at 0.6 mm full width at half maximum and separation at 0.4 mm. Additionally, Gaussian smoothing was applied with a full width at half maximum of 0.8 mm.

The processing step involved aligning the data to a rat atlas, followed by segmentation and statistical analysis. To achieve registration and segmentation, all images were initially aligned and registered to a 3D Rat Brain Atlas©, which included 173 segmented and annotated brain regions. This alignment was performed using the GUI-based EVA software developed by Ekam Solutions (Boston, MA). The image registration process encompassed translation, rotation and scaling adjustments, performed independently in all three dimensions. All spatial transformations applied were compiled into a matrix [Tj] for each subject. Each transformed anatomical pixel location was tagged with its corresponding brain area, resulting in fully segmented representations of individual subjects within the atlas.

Each scanning session consisted of 350 data acquisitions [number of repetitions (NR)] with a period of 6 s (repetition time) each for a total lapse time of 35 min. The first 50 scans (5 min) were control window, while the stimulation window was 51–350 (for 30-min post-injection) scans. Statistical *t*-tests were performed on each voxel (∼36 000 in number of voxels in the whole brain) of each subject within their original coordinate system. The baseline threshold was set at 1%. The *t*-test statistics used a 95% confidence level (*P* < 0.05), two-tailed distributions and heteroscedastic variance assumptions. As a result of the multiple *t*-test analyses performed, a false-positive detection controlling mechanism was introduced. This subsequent filter guaranteed that, on average, the false-positive detection rate was below our cut-off of 0.05. The formulation of the filter satisfied the following expression:


$$P_{i}\leq \;\frac{i}{V}\frac{q}{c(V)}\;.$$

In the equation, *P*_i_ represents the *P*-value derived from the *t*-test conducted at the *i*-th pixel within the region of interest, comprising V pixels, with each pixel ranked according to its probability value. For our analysis, we set the false-positive filter value *q* at 0.2, and we fixed the predetermined constant *c*(*V*) at unity, following a conservative approach for assessing significance.^17^ Pixels that achieved statistical significance retained their relative percentage change values, while all other pixel values were set to zero. Our analysis employed a 95% confidence level, two-tailed distributions and assumed heteroscedastic variance for the *t*-tests.

To create composite maps displaying the per cent changes in the BOLD signal for each experimental group, we mapped each composite pixel location (in terms of rows, columns and slices) to a voxel within the *j*-th subject using the inverse transformation matrix [Tj]-1. A trilinear interpolation method was used to determine the contribution of subject-specific voxel values to the composite representation. The use of inverse matrices ensured that the entire composite volume was populated with subject inputs. The average of all contributions was assigned as the per cent change in the BOLD signal at each voxel within the composite representation of the brain for the respective experimental group.

**Figure 1:**
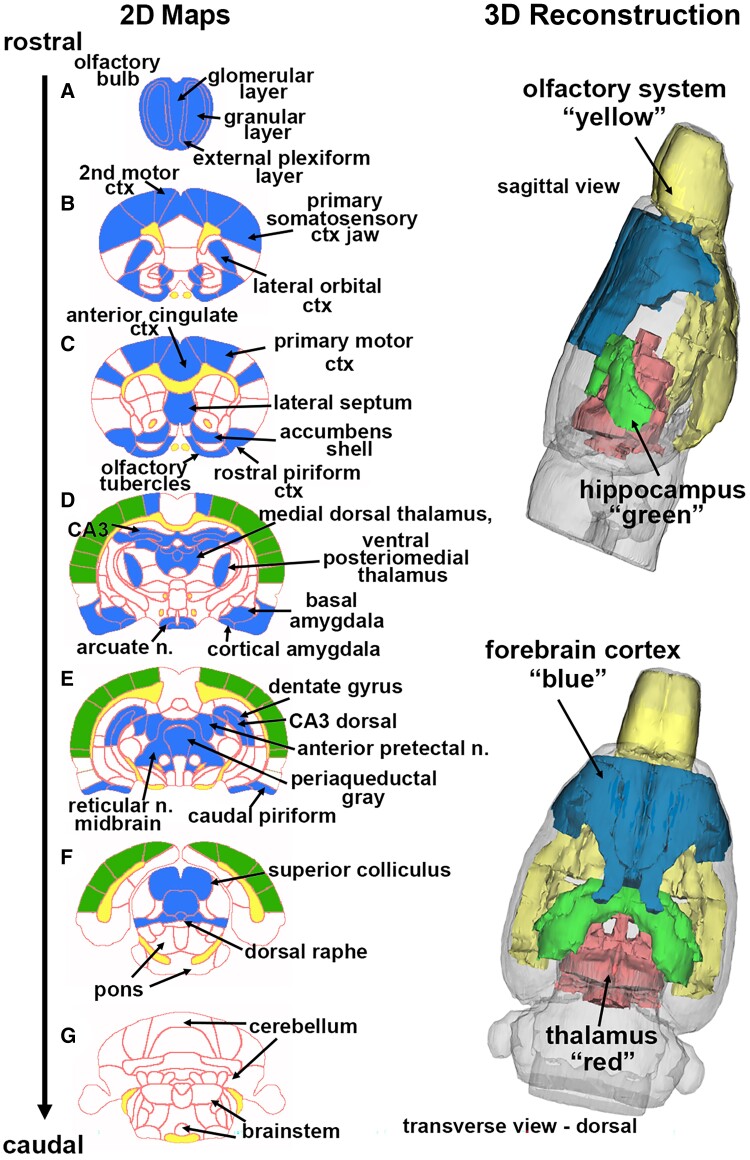
**Change in BOLD signal with high-dose LSD.** Depicted are 2D coronal sections **A–G** showing the location of brain areas that were significantly different in negative BOLD signal (blue highlight) between Veh and high-dose (100 *µ*g/kg) LSD. White matter tracts are highlighted in yellow. The primary SS, auditory and visual cortices that were not significantly affected are shown in green. The 3D colour-coded reconstructions summarize the major brain areas that were significantly different. n., nucleus.

In the post-processing phase, we compared the number of activated voxels in each of the 173 brain regions between the control and LSD doses using a Kruskal–Wallis test statistic. The data were ranked in order of significance, as detailed in **Tables 1–4**. We generated activation maps, depicted in Fig. 1, showing brain areas with significant differences when comparing two or more groups.

**Table 1:** Positive volume of activation: Veh versus 10 *µ*g/kg LSD

	Veh		10 *µ*g/kg LSD	
Brain area	Med		Med	*P*-value
Supra-optic n.	1	>	0	0001
Substantia innominata	2	>	0	0.027
Granular cell layer	181	>	46	0.027
Glomerular layer	116	>	44	0.034
Medial amygdala	22	>	6	0.045
External plexiform layer	62	>	17	0.05
Caudal piriform ctx	109	>	73	0.05

**Table 2:** Negative volume of activation: Veh versus 10 *µ*g/kg LSD

	Veh		10 *µ*g/kg LSD	
Brain area	Med		Med	*P*-value
Olfactory tubercles	5	<	63	0.005
Basal amygdala	7	<	25	0.007
Subthalamic n.	1	>	0	0.007
Caudal piriform ctx	38	<	107	0.009
Secondary motor ctx	5	<	30	0.01
Lateral posterior thalamus	0	<	8	0.012
Substantia nigra reticularis	3	<	13	0.015
Cortical amygdala	4	<	19	0.017
88frontal association ctx	1	<	25	0.02
Perirhinal ctx	37	<	65	0.025
Magnocellular preoptic n.	0	<	2	0.029
Intercalated amygdala	0	<	1	0.032
Medial amygdala	2	<	9	0.033
Paramedian lobule	14	<	53	0.041
Primary motor ctx	6	<	52	0.041
Ventral pallidum	16	<	38	0.041
Rostral piriform ctx	34	<	99	0.041
Superior colliculus	47	<	81	0.041

**Table 3:** Positive volume of activation: Veh versus 100 *µ*g/kg LSD

	Veh		100 *µ*g/kg LSD	
Brain area	Med		Med	*P*-value
Pontine nuclei	12	<	44	0.005
Caudal piriform ctx	109	>	44	0.008
External plexiform layer	62	>	25	0.012
Medial cerebellar n. fastigial	0	>	4	0.017
Simple lobule cerebellum	3	>	36	0.017
Glomerular layer	116	>	36	0.018
Cochlear n.	2	<	14	0.019
Substantia innominata	2	>	0	0.030
Granular cell layer	181	>	81	0.035
Lateral geniculate	10	>	0	0.037
Interposed n.	0	<	4	0.041
Lateral amygdaloid n.	5	>	0	0.047
Primary motor ctx	216	>	71	0.048

**Table 4:** Negative volume of activation: Veh versus 100 *µ*g/kg LSD

	Veh		100 *µ*g/kg LSD	
Brain area	Med		Med	*P*-value
Medial dorsal n. thalamus	0	<	10	0.002
Caudal piriform ctx	39	<	99	0.002
Primary SS ctx jaw	6	<	88	0.005
Cortical amygdala	4	<	30	0.005
Habenula n. thalamus	0	<	8	0.006
CA3 dorsal	6	<	58	0.007
Lateral posterior n. thalamus	0	<	37	0.007
Basal amygdala	7	<	39	0.008
Superior colliculus	47	<	95	0.008
Retrochiasmatic n.	0	<	1	0.012
Lateral dorsal n. thalamus	0	<	1	0.012
Olfactory tubercles	5	<	56	0.015
Rostral piriform ctx	34	<	111	0.015
Lateral septal n.	7	<	54	0.022
Accumbens shell	5	<	40	0.023
Primary motor ctx	6	<	79	0.023
Premammillary n.	0	<	1	0.025
Dorsal raphe	0	<	5	0.026
Paraventricular n. thalamus	0	<	2	0.032
Frontal association ctx	1	<	10	0.033
Anterior pretectal n. thalamus	0	<	3	0.034
Reticular n. midbrain	14	<	122	0.035
Secondary motor ctx	5	<	28	0.037
Ventral posteromedial n. thalamus	7	<	39	0.040
Lateral orbital ctx	6	<	50	0.041
Dentate gyrus dorsal	16	<	43	0.041
Granular cell layer	32	<	84	0.044
External plexiform layer	9	<	35	0.044
Glomerular layer	25	<	49	0.044
Periaqueductal grey	39	<	70	0.044
Anterior cingulate area	4	<	37	0.047
Arcuate n.	0	<	2	0.047

### Resting-state functional connectivity

#### Image acquisition

Scans were collected using a spin-echo triple-shot echo-planar imaging sequence [imaging parameters: matrix size = 96 × 96 × 20 (H × W × D), repetition time/TE = 1000/15 ms, voxel size = 0.312 × 0.312 × 1.2 mm, slice thickness = 1.2 mm, with 200 repetitions, and time of acquisition 15 min]. For pre-processing, we utilized a combination of various software tools, including Analysis of Functional NeuroImages (AFNI_17.1.12), the FMRIB Software Library (FSL, v5.0.9), Deformable Registration via Attribute Matching and Mutual-Saliency Weighting (DRAMMS 1.4.1) and MATLAB. Brain tissue masks for rsFC images were manually delineated using 3DSlicer and applied for skull stripping. We identified motion outliers, which are data segments affected by substantial motion, and recorded the corresponding time points for later regression. Large motion spikes were also detected and removed from the time course signals. Following this step, slice timing correction was applied to account for interleaved slice acquisition order. We performed head motion correction using the six motion parameters, with the first volume serving as the reference image. Normalization involved registering functional data to the 3D MRI Rat Brain Atlas© using affine registration through DRAMMS. This atlas contains 173 annotated brain regions and was used for segmentation. After quality control, a band-pass filter (0.01–0.1 Hz) was applied to reduce low-frequency drift effects and high-frequency physiological noise for each subject. The resulting images underwent detrending and spatial smoothing, with a full width at half maximum of 0.8 mm. Additionally, regressors, including motion outliers, the six motion parameters, the mean white matter and cerebrospinal fluid time series, were incorporated into general linear models for nuisance regression to eliminate unwanted effects.

The region-to-region functional connectivity analysis was conducted to measure the correlations in spontaneous BOLD fluctuations. In this analysis, a network consists of nodes (brain regions of interest) and edges (connections between regions). We averaged the voxel time series data within each node based on the residual images obtained through the nuisance regression procedure. Pearson’s correlation coefficients were computed across all pairs of nodes (14 535 pairs) for each subject within all three groups to assess interregional temporal correlations. The resulting *r*-values, ranging from −1 to 1, were z-transformed using Fisher’s Z transform to improve their normality. We constructed 166 × 166 symmetric connectivity matrices, with each entry representing the strength of an edge. Group-level analysis was then conducted to examine functional connectivity in the experimental groups. The *Z*-score matrices obtained from one-group *t*-tests were clustered using the K-nearest neighbour clustering method to identify how nodes cluster together and form resting-state networks. A *Z*-score threshold of |Z| = 2.3 was applied to eliminate spurious or weak node connections for visualization purposes.

### Functional connectivity analysis

#### Degree centrality

We conducted all network analysis using Gephi, which is an open-source software for network analysis and visualization.^18^ We imported the absolute values of the symmetric connectivity matrices for both LSD and Veh data, treating the edges as undirected networks. Degree centrality analysis measures the number of connections that a particular node has within the entire network. Degree centrality is defined as:


$$C_{D}(j)=\overset{n}{\underset{\;j=1}{\sum }}\;A_{\mathrm{ij}}.$$

Here, ‘*n*’ represents the total number of rows in the adjacency matrix denoted as ‘*A*’, and the individual elements of the matrix are indicated as ‘*A*_ij_’, which signifies the count of edges connecting nodes *i* and *j*.

### Statistics

We conducted all statistical analysis for the graph theory assessment using GraphPad Prism. To decide whether parametric or non-parametric assumptions were appropriate for different group subregions, we performed normality tests. We used Shapiro–Wilk’s tests to assess the normality assumption. Subregion degree centrality *P* > 0.05 was considered to exhibit a normal distribution. Once the normality assumptions were confirmed, we employed paired *t*-tests to compare the degree centrality between the experimental groups in various subregions. In cases where there was evidence against the normality assumption, we conducted a non-parametric Wilcoxon signed-rank test.

## Results

**Tables 1** (positive BOLD) and 2 (negative BOLD) are lists of brain areas showing the median (Med) number of significant voxels between Veh and the low dose (10 *µ*g/kg) of LSD. The brain areas are ranked in order of their significance using a critical value of *P* < 0.05. For positive BOLD, only 7/173 brain areas showed a significant difference in the volume of activation (i.e. voxel number), in response to the low-dose LSD. In all cases, there was a decrease in positive BOLD. With a false discovery rate (FDR) of *P* = 0.008, the low-dose LSD-induced change in positive BOLD would be deemed insignificant; however, note the representation of the olfactory system, e.g. granular, glomerular and external plexiform layers of the olfactory bulb and the piriform cortex. As seen in **Table 2**, the LSD-induced negative BOLD affected 18/173 brain areas (FDR *P* = 0.021). In all cases with the exception of the subthalamic nucleus (n.), there was an increase in negative BOLD. Again the olfactory system is represented by the piriform cortices, olfactory tubercles and cortical amygdala. The amygdaloid region was represented by the basal, medial and intercalated nuclei. The decrease in positive BOLD and increase in negative BOLD would suggest low-dose LSD is reducing activity in these brain areas. Tables for all 173 brain areas, negative and positive volume of activation, are provided in Supplementary Data 1.

**Tables 3** and **4** are lists of areas that are significantly different between Veh and high-dose LSD (100 *µ*g/kg). The brain areas are ranked in order of their significance using a critical value of *P* < 0.05. For positive BOLD, 13/173 brain areas showed a significant difference in the volume of activation in response to high-dose LSD (FDR *P* = 0.015). All areas showed a decrease in positive BOLD voxels with the exception of the pontine n., cochlear n. and the interposed n. of the cerebellum. The high-dose LSD also targeted the olfactory system. The olfactory bulb (e.g. granular, glomerular and plexiform layers) and olfactory cortex (e.g. caudal piriform) all showed reduced positive BOLD. As seen in **Table 4**, the LSD-induced negative BOLD affected 32/173 brain areas (FDR *P* = 0.037). In all cases, there was an increase in negative BOLD. In addition to the olfactory system, there were several thalamic nuclei that were affected. Going from 10 to 100 *µ*g/kg LSD, there was a similar decrease in putative brain activity but over a greater number of brain areas. Tables for all 173 brain areas, negative and positive volume of activation, are provided in Supplementary Data 1.

Figure 1 shows the anatomical localization of the brain areas listed in **Table 4** presented as 2D activation maps. The coronal sections are labelled **A–G** and arranged from rostral (*top*) to caudal (bottom). Areas in blue are significantly different between Veh and 100 *µ*g/kg LSD. Areas in yellow denote the location of white matter tracts. Figure 1A shows the olfactory bulb with the three different layers. Figure 1B and C shows forebrain cortical areas, e.g. second and primary motor cortices, anterior cingulate and lateral orbit cortices and the most rostral area of the somatosensory (SS) cortex representing the jaw. Figure 1C highlights the accumbens shell and olfactory tubercles, brain areas with dopaminergic afferent connections from ventral tegmental area, while the lateral septum has direct efferent connections to the ventral tegmental area. Figure 1D and E highlights the dorsal hippocampus (e.g. CA3 and dentate gyrus), amygdala and numerous thalamic nuclei. After the forebrain (Fig. 1A–C), there is no effect of high-dose LSD on the SS cortex, including areas representing the trunk, shoulder, limbs, barrel field and lips including the auditory and visual cortices. These areas are not annotated but highlighted in green. Also, note that the caudal end of the brain represented by brainstem, cerebellum and pons is unresponsive to high-dose LSD. The 3D reconstructions in Fig. 1 summarizes the major brain areas affected by high-dose LSD.

**Figure 2:**
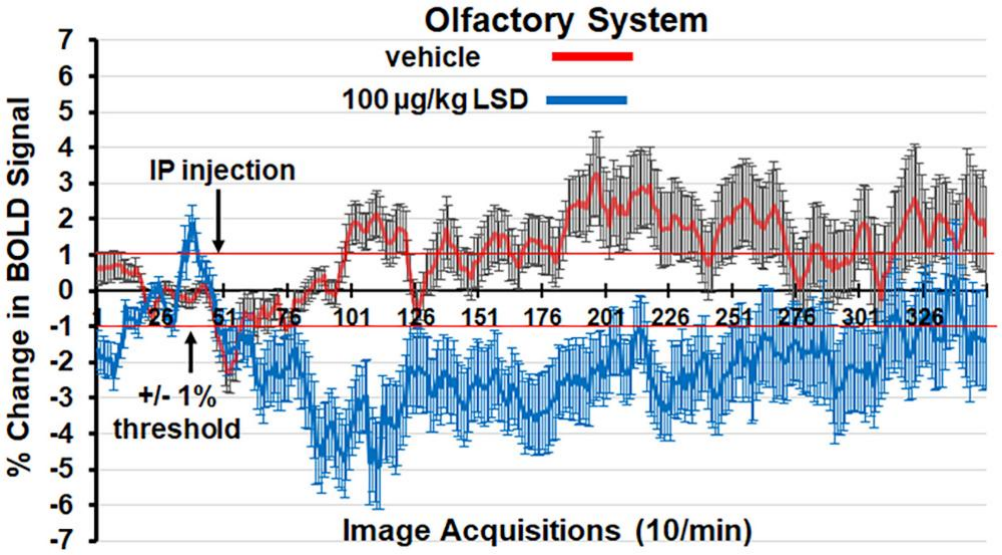
**Change in BOLD signal over time.** Shown is the change in BOLD signal over time for the olfactory system in response to Veh (red line) and 100 *µ*g/kg of LSD (blue line). Three hundred fifty images were acquired over the 35-min imaging session. Each acquisition is the mean ± Se of Veh and high-dose LSD rats combining the data from the three layers of the olfactory bulb, rostral and caudal piriform cortex and olfactory tubercles for all subjects for each experimental condition. The 1% threshold is highlighted by the red lines to account for the normal fluctuations in BOLD signal in the awake rat brain. A two-way ANOVA showed a significant time × treatment interaction, *F* = 4.424, *P* < 0.0001. IP, intraperitoneal.

Figure 2 shows the change in BOLD signal over time for the olfactory system in response to Veh (red line) and 100 *µ*g/kg of LSD (blue line). Three hundred fifty images were acquired over the 35-min imaging session. Each acquisition is the mean ± Se of Veh and high-dose LSD rats combining the data from the three layers of the olfactory bulb, rostral and caudal piriform cortex and olfactory tubercles for all subjects for each experimental condition. With a two-way repeated measures ANOVA, there was a significant interaction between time and treatment [*F*_(249.20242)_ = 4.424, *P* < 0.0001]. Note the decrease in BOLD signal following IP injection (image acquisition # 50) of LSD. Indeed, by 5-min post-injection of LSD (acquisition # 100), there was a 4% decrease in BOLD signal that remained below threshold (thin red line) for almost the entire imaging session. In contrast, Veh injection was characterized by a slow increase in positive BOLD signal that ranged between 1 and 2% following 30-min post-injection.

**Figure 3:**
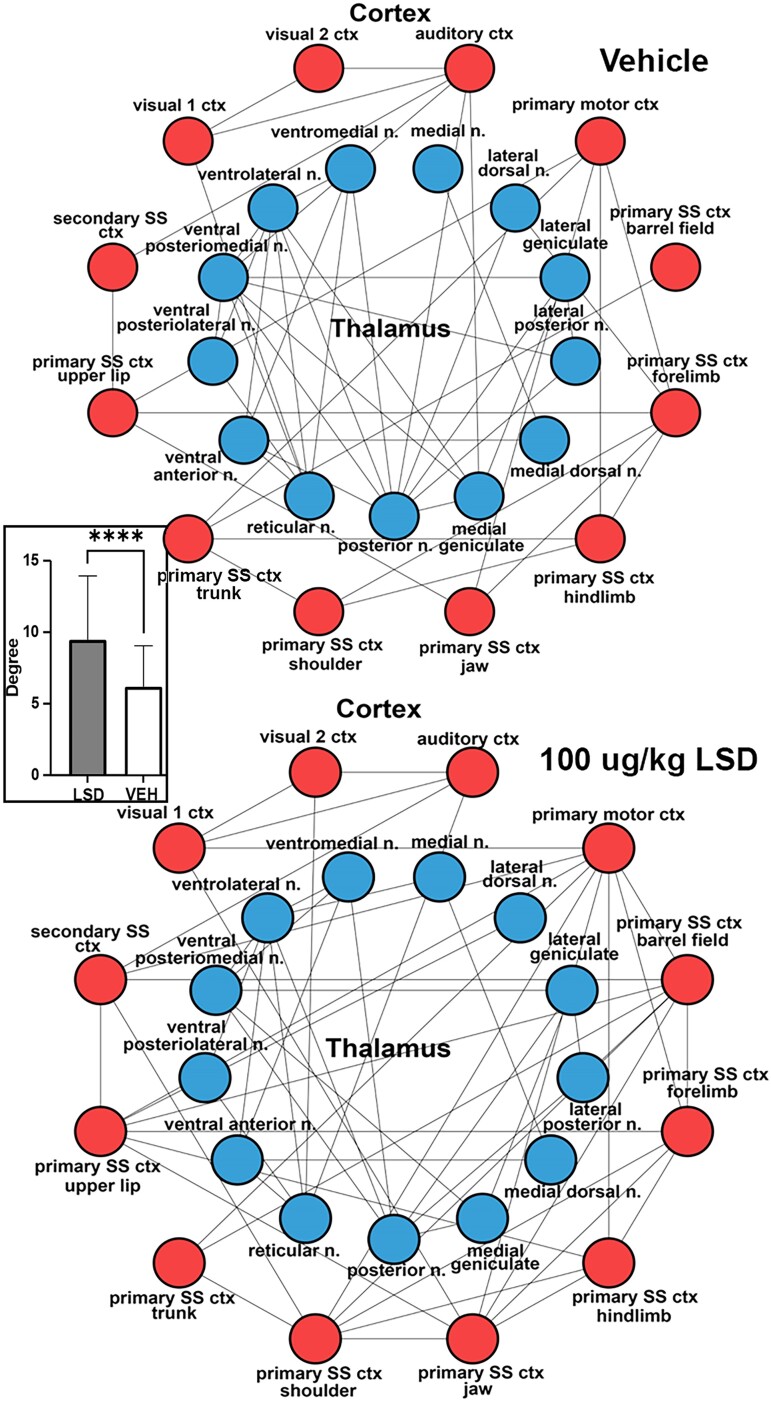
**Thalamo-cortical connectivity.** Shown are radial representations of the connections or degrees (lines) between thalamic nuclei (blue circles) and SS, visual and auditory cortices (red) for Veh and LSD treatments. The insert are bar graphs (mean ± Sd) for the number of degrees between LSD and Veh. Student’s *t*-test, ****P < 0.0001. SS, somatosensory.

In Fig. 3 the rsFC, i.e. the nodes (brain areas) and edges (lines) connecting the different thalamic (blue circles) and sensory and motor cortices (red circles), are depicted as radial wheels for the Veh and 100 *µ*g/kg dose of LSD. The cortex was limited to primary SS and motor cortices including vision and audition. The limbic cortex was not included. Only specific sensory and motor relay nuclei were included in the thalamus. For example, lateral geniculate is specific for vision, medial geniculate for hearing and the different ventral and dorsal posterior nuclei for somatosensation. The ventral anterior and ventral lateral thalamic nuclei are involved in motor control and project to the motor cortex. The rsFC data were collected ca 45 min following LSD treatment. There were significantly more corticothalamic connections or degrees following exposure to LSD. This difference is shown in the inserted bar graph.

**Figure 4:**
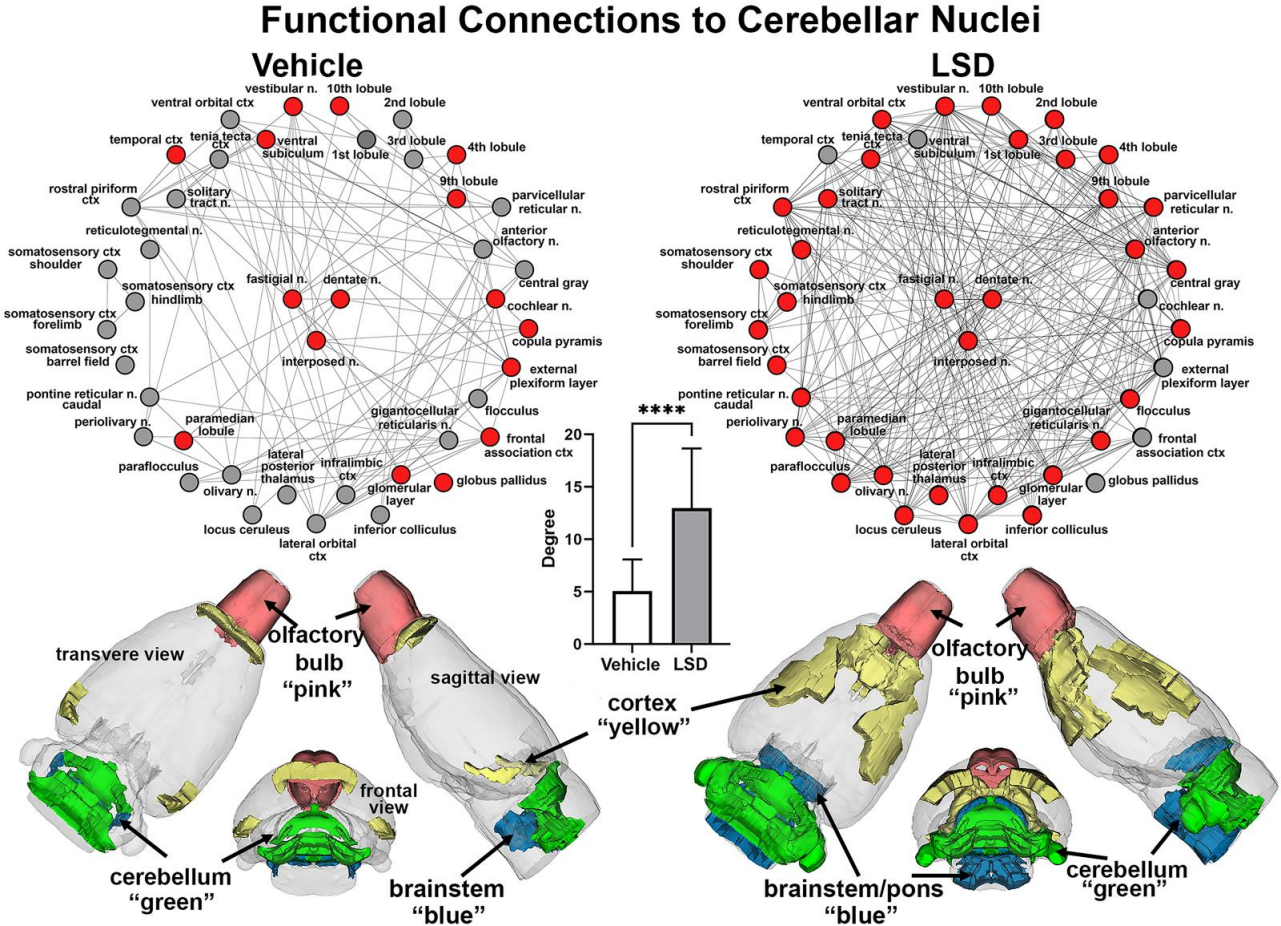
**Cerebellar connectivity.** Shown are radial representations of the connections or degrees (lines) to the three cerebellar nuclei (centre red), e.g. fastigial, dentate and interposed following Veh or 100 *µ*g/kg LSD treatment. The total network is represented by 39 brain areas as the union of both treatments. All brain areas highlighted in red have direct connections to the cerebellar nuclei under each experimental condition. The significant difference in connectivity (Student’s *t*-test, ****P < 0.0001) is shown in the inserted bar graph at the centre of the figure. The 39 brain areas comprising the network can be organized in four major brain regions—cortex, olfactory bulbs, cerebellum and brainstem/pons. The 3D colour-coded reconstructions show the location of these brain regions for Veh and LSD.

Figure 4 shows the functional connectivity to the three cerebellar nuclei (centre, red), e.g. fastigial, dentate and interposed following Veh or 100 *µ*g/kg LSD treatment. The total network is represented by 39 brain areas as the union of both treatments. All brain areas highlighted in red have direct connections to the cerebellar nuclei under each experimental condition. Compared to Veh, rats exposed to LSD present with hyperconnectivity (33/39 nodes) to cerebellar nuclei, while Veh controls show only 13/39 nodes with direct connections to these nuclei. The significant difference in connectivity (*P* < 0.0001) is shown in the inserted bar graph at the centre of Fig. 4. It should be noted that many of the brain areas with direct connections to the cerebellar nuclei with Veh, e.g. temporal cortex, ventral subiculum, cochlear n., external plexiform layer, frontal association ctx and globus pallidus loss their connections with LSD exposure. The 39 brain areas comprising the network can be organized in four major brain regions—cortex, olfactory bulbs, cerebellum and brainstem/pons. The 3D colour-coded reconstructions show the location of these brain regions for Veh and LSD.

## Discussion

This study followed dose-dependent changes in brain activity in response to LSD. Male and female rats were drug-naive and fully awake when exposed to LSD during the scanning session. There were no significant differences between male and female rats. To our knowledge, these are the first preclinical functional MRI studies done on this drug. The data were registered to a rat MRI atlas with 173 3D brain areas providing a global map of site-specific changes. While it is well established that LSD’s hallucinogenic effects involve serotonin 5-HT_2A_ receptor activation^19^ it is a promiscuous molecule affecting several other signalling system, e.g. dopamine receptors D_2/3_, mechanistic target of rapamycin, tropomyosin receptor kinase and trace amine-associated receptor 1.^8-10^ phMRI provides an agnostic picture of brain activity independent of cellular mechanism.^14,20,21^ Brain activity in awake rodent imaging is characterized by the presence of both positive and negative BOLD signal following Veh or drug administration. The positive BOLD reflects an increase in neuronal activity accompanied by an increase in blood flow while the negative BOLD a decrease in brain activity and reduction in blood flow.^22,23^ In these studies, the predominant acute effect of LSD on BOLD signal was a decrease in positive BOLD and increase in negative BOLD to specific regions of the brain. This effect is discussed in the context of previous preclinical behavioural studies and, more importantly, with respect to the human imaging data.

### Rodent studies

The reduction in positive BOLD and increase in negative BOLD would suggest a reduction in neuronal activity that would be accompanied by a decrease in behaviour. The pronounced reduction in activity of the olfactory system, something not noted in the animal literature, would have consequential effects on behaviour as rodents primarily interact with their environment through their sense of smell.^24^ There are numerous studies in rodents reporting LSD reduces behavioural activity. For example, a single dose of LSD in doses ranging from 5–200 *µ*g/kg reduces locomotor activity shortly after treatment.^25-29^ LSD given as a single dose of 50–100 *µ*g/kg to adult male mice is reported to reduce ethanol consumption and binge drinking.^30,31^ LSD given in doses of 60 or 240 *µ*g/kg to awake rats reduce the communication between the visual cortex and hippocampal activation of CA1 place neurons while creating a state of immobilization for 30-min post-treatment with recurring high voltage spikes that are characteristic of the transition between wakefulness-to-sleep.^32^ The prolonged immobility for 30 min together with the loss of communication between hippocampus place neurons and visual cortex would suggest a dissociation in perception. It would be interesting to know how much of the disruption in rodent behaviour is caused by the putative loss of smell suggested in this study.

The enhanced dose-dependent negative BOLD maybe explained, in part, by the LSD-induced dose-dependent decrease in serotonergic and dopaminergic activity reported by De Gregorio and coworkers.^9^ Adult male rats given i.v. LSD in doses ranging from 30–120 *µ*g/kg show a decrease in firing rates of serotonergic neurons in the dorsal raphe and dopaminergic neurons in the ventral tegmental area. The reduced activity in these systems to the acute presentation of LSD is reversed with chronic exposure of drug. Male mice treated once daily for seven consecutive days with low-dose LSD (30 *µ*g/kg) show enhanced social behaviour an effect attributed to 5-HT_2A_ and AMPA receptor activation in the prefrontal cortex mediated by an mTORC1 pathway in glutamatergic excitatory neurons.^33^

### Human imaging studies

The pronounced dose-dependent negative BOLD was unexpected. There have been several studies testing LSD on brain function in healthy volunteers using MRI.^4-6^ However, these studies gave LSD or Veh outside the magnet, 1–2 h prior to imaging. During the imaging session, LSD-treated subjects are recorded for resting functional connectivity or exposed to different cognitive and behavioural provocations and the BOLD signal changes compared to control subjects. To the best of our knowledge, no one has imaged the acute effects of LSD on drug-naive healthy volunteers. So the reported dose-dependent negative BOLD in our study is without precedent in the animal or human literature. However, 45-min post-exposure to LSD, there are significant changes in rsFC that complement those reported in the human literature. For example, we noted a significant increase in connectivity between thalamus and sensorimotor cortices with the high dose of LSD.^4-6,34^ A recent study by Delli Pizzi and colleagues^35^ using a seed-based approach identified the functional connections between select thalamic nuclei, e.g. pulvinar and the ventral lateral and ventral posterior complex, and SS and auditory cortex that were sensitive to LSD in healthy volunteers. They propose these connections are driven by LSD activation of 5-HT_2A_ receptors in the cortex. Effective connectivity or the causal modelling of functional MRI data shows LSD enhances effective connectivity from thalamus to unimodal cortices, e.g. auditory and visual, but reduces the top-down connectivity of cortex to thalamus.^36^ LSD also increases effective connectivity from thalamus to cingulate cortex but decreases connectivity from striatum to thalamus affecting the cortico-striato-thalamo-cortical loop involved in information processing and thalamo-cortical gaiting of sensory information^37^ A recent study by Bedford and coworkers^38^ reported changes in whole brain connectivity and effective connectivity in healthy volunteers following treatment with LSD. LSD caused similar increase in global and effective connectivity with effective connectivity highlighting a decrease in connectivity between the bilateral occipital cortices and the bilateral putamen and cerebellum. It should be noted that the reduction in connectivity between the putamen and cerebellum is the only example in the literature of LSD affecting cerebellar function of which we are aware. While many studies have emphasized the role of serotonin as mediating the effects of LSD on behaviour, a recent report by Grazer and colleagues^39^ have focused attention on the dopaminergic system. They used EEG to follow event-related potentials related to reward processing in healthy volunteers given low doses of LSD. The changes in EEG signals suggested low doses of LSD increase sensitivity in the dopaminergic reward system.

### Cerebellum

We were not surprised to see the involvement of the cerebellum given the many awake animal imaging studies showing changes in cerebellar activity following treatment with cannabinoids, psychostimulants, ketamine, opioids and even neuropeptides.^14,21,40-42^ Traditionally, the cerebellum is viewed as a brain region primarily associated with motor coordination. However, it participates in autonomic physiological processes such as heart rate, blood pressure and respiration.^43-46^ The cerebellum is recognized as having a significant role in emotional and cognitive functions,^47-49^ feeding^50^ and addiction.^51^ The cerebellar nuclei have extensive reciprocal connections to many brain regions including the thalamus, hypothalamus, limbic cortex, amygdala, hippocampus and brainstem.^52^ Notably, the cerebellum not only exerts efferent control over autonomic function but is also directly influenced by visceral afferents through the splanchnic and vagus nerves.^52^ The cerebellum receives much of its innervation from the vestibular complex, the brain area relaying auditory information from the ear to the cortex. This expansive connectivity and array of behavioural and sensory functions raises the question—does the cerebellum contribute to the psychotomimetic effects of hallucinogens? Interestingly, given the role of the cerebellum in various cognitive, emotional and sensory functions, it could contribute to the diverse array of symptoms and cognitive dysfunctions seen in schizophrenia, a notion promoted by Andreasen and colleagues.^53^ The hyperconnectivity to the cerebellar nuclei noted in this study in response to LSD was localized to specific brain regions. The prefrontal cortex together with the SS cortices were recruited in addition to several areas in the brainstem/pons that form the ascending reticular activating system e.g. gigantocellularis, locus coeruleus and parvocellular reticular n.

### Data interpretation and limitations

While phMRI gives a global picture of changes in brain activity, it does not address mechanism. How much of the data in this study would have been attributed to LSD interacting with 5-HT_2A_ or D_2_ receptors? Preller and colleagues^6^ reported the thalamic connectivity in altered perception was mediated by 5-HT_2A_ receptors. Using radiolabelled raclopride, a high-affinity D_2_ receptor antagonist with PET imaging in a pig, Minuzzi *et al*.^54^ clearly showed ligand displacement in the striatum with LSD treatment. Treatment with a 5-HT_2A_ or D_2_ receptor antagonist would have helped parse out the contribution made by each of these receptors in the BOLD signal and site-specific patterns of activation. While the doses of LSD used in these studies were drawn from the studies by De Gregorio *et al*.,^9^ we did not assay blood and brain for levels of LSD.

Do these data provide insight into the psychedelic effects of LSD? The high dose of LSD in our study (100 *µ*g/kg) is reported to impair sensorimotor gating and cause head twitching, behaviours presumably associated with hallucinations.^55-57^ Disruption in thalamic filtering of efferent sensory information to specific sensorimotor/unimodal cortices (e.g. audition and vision) are hypothesized to alter perception and contribute to hallucinations.^5,37^ In this study, there is an absence of behavioural data to confirm head twitching and reduced motor behaviour.

## Conclusion

There was an acute dose-dependent decrease in brain activity with LSD treatment as surmised by an increase in negative BOLD signal. The areas most affected were the primary olfactory system, prefrontal cortex, thalamus and hippocampus. This would suggest a general decrease in neuronal activity and behaviour as reported in the literature. By 45-min post-treatment, there was an increase in functional connectivity between thalamus and sensorimotor cortices as would be predicted from clinical studies. However, in addition, there was an increase in functional connectivity to the cerebellar nuclei, a finding not reported in clinical studies on LSD.

## Supplementary Material

fcae194_Supplementary_Data

## Data Availability

The data that support the findings of this study are available from the corresponding author, on request.
